# Cross-Domain Traffic Scene Understanding by Integrating Deep Learning and Topic Model

**DOI:** 10.1155/2022/8884669

**Published:** 2022-03-18

**Authors:** Yuanfeng Yang, Husheng Dong, Gang Liu, Liang Zhang, Lin Li

**Affiliations:** ^1^Jiangsu Province Support Software Engineering R&D Center for Modern Information Technology Application in Enterprise, Suzhou, China; ^2^School of Computer Engineering, Suzhou Vocational University, Suzhou, China; ^3^School of Computer and Information Engineering, Xiamen University of Technology, Xiamen, China

## Abstract

Understanding cross-domain traffic scenarios from multicamera surveillance network is important for environmental perception. Most of existing methods select the source domain which is most similar to the target domain by comparing entire domains for cross-domain similarity and then transferring the motion model learned in the source domain to the target domain. The cross-domain similarity between overall different scenarios with similar local layouts is usually not utilized to improve any automatic surveillance tasks. However, these local commonalities, which may be shared across multiple traffic scenarios, can be transferred across scenarios as prior knowledge. To address these issues, we present a novel framework for cross-domain traffic scene understanding by integrating deep learning and topic model. This framework leverages the labeled samples with activity attribute labels from the source domain to annotate the target domain, where each label represents the local activity of some objects in the scene. When labeling the activity attributes of the target domain, there is no need to select the source domain, which avoids the phenomenon of performance degradation or even negative transfer due to wrong source domain selection. The effectiveness of the proposed framework is verified by extensive experiments carried out using public road traffic data.

## 1. Introduction

The tremendous development of mobile Internet of Things (IoT) has led to the widespread deployment of cooperative vehicle–infrastructure systems (CVISs) based on Internet of Vehicle (IoV) networks, promoting the development of intelligent transportation [1]. Vehicle–infrastructure cooperative data communication requires high-speed, stable, and low-latency communication technology as a guarantee. Xu et al. [2–5] ensure efficient and stable communication by predicting the outage probability (OP). With the rapid development of computer vision technology, intelligent video surveillance system has become an important part of intelligent transportation system (ITS). Through video sensor nodes, the system can capture the visual appearance of moving objects timely and accurately and extract more information about them through object detection, tracking, behavior analysis, and so on. The widespread use of intelligent video surveillance systems on road networks has generated an unprecedented amount of surveillance data. Numerous studies have been motivated to adopt automated means to model, understand, and exploit these data. Scene understanding/behavior analysis is one of the key tasks of automatic surveillance video understanding, which reveals the typical activities and behaviors in surveillance scenarios [6–9]. So far, this task is generally to train and deploy models within a single scene or a group of adjacent/similar scenarios. However, it is not scalable to annotate training examples in each scene exhaustively, which leads to the fact that the trained model cannot be readily applied across domain.

**Figure 1:**
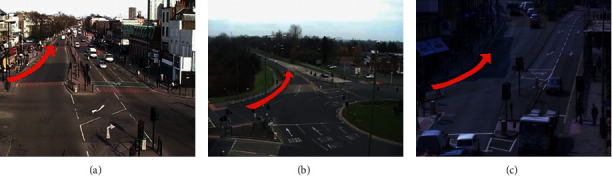
Typical motion patterns in different scenarios, e.g., Left2Up Turn. These patterns (red arrows) share similar semantic content: turning from left to up.

To address this problem, several approaches have been proposed [10–12]. The common denominator of these methods is to select the most similar/relevant source domain to the target domain by comparing the cross-domain similarity between the entire domains and then transfer the motion model learned from the source domain to the target domain. However, inappropriate source domain selection can lead to performance degradation or even negative transfer. More critically, considering only the cross-domain similarity between the entire domains ignores the cross-domain similarity between scenarios that are different but locally relevant (e.g., many different traffic scenarios with similar local layouts), which can be used to improve automatic monitoring tasks. As shown in Figure 1, the local motion patterns (indicated by red arrows) in different traffic scenarios share similar semantic content: turning from left to up (Left2Up Turn). Since these typical patterns can be shared across multiple traffic scenarios, we can leverage the local prior knowledge obtained from source domain scenarios to interpret a new target domain scene unsupervised.

Human beings have outstanding performance in identifying unseen objects through the attribute knowledge of empirical objects [13, 14]. For example, when a red light is on at a road intersection, drivers can tell that it is a no-passing sign based on their existing knowledge, even if they have not been in the scene before. In traffic scenarios, moving objects follow specific motion patterns (Left2Up Turn, Up2Left Turn, etc.) governed by traffic rules. If all these motion patterns are considered as a set of activity attributes, each surveillance video clip may be labeled with multiple activity attributes (subset). This issue can then be viewed as a multilabel classification problem [15–17]. By allowing these attribute labels obtained from the source domain scenarios to be propagated to the new target domain scenarios, cross-domain application problems may be solved.

**Figure 2:**
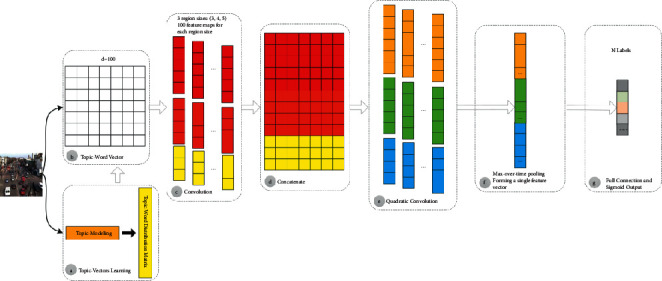
An overview of the proposed framework for cross-domain traffic scene understanding.

In this study, we develop a new framework for cross-domain traffic scene understanding illustrated in Figure 2. We first combine the Word2Vec word vector model and the LDA topic model to construct the topic-word vector matrix, which takes into account both word granularity level features and text granularity level semantic features representation. Then, the text convolutional neural networks (TextCNN) with two superimposed convolutional layers are used to obtain the joint features from the constructed topic-word vector matrix, and the acquired features are sent to the sigmoid classifier to get the classification results. In addition to transforming scene understanding into a multilabel classification problem with activity attributes, we align two scenarios (source domain scene and target domain scene) using geometric transformations including scaling and translation and feed the transformed topic-word vector matrix of the target domain scene into TextCNN mentioned above for multilabel classification.

The main contributions of this study are as follows:

1. We formulate a new idea to solve the cross-domain traffic scene understanding problem, which is to transform this problem into a multilabel classification problem with activity attributes (local motion patterns) for solving.
2. We introduce a scheme for transferring the local prior knowledge obtained from source domain scenarios to a new target domain scene, avoiding the phenomenon of performance degradation or even negative transfer due to wrong source domain selection.
3. We employ a topic-word vector matrix transformation strategy, which is executed before the topic-word vector matrix of the target domain scene is input into TextCNN for multilabel classification to reduce the cross-scene variance.

The remaining of this paper is organized as follows: the related work is discussed in Section 2.  Section 3 describes the architecture of the proposed framework integrating TextCNN with LDA topic model. Similarity measurement for scene activities and scene alignment is introduced in Section 4.  Section 5 describes the experimental setup and results. Finally, we draw conclusions in Section 6.

## 2. Related Works

### 2.1. Behavior Analysis for Scene Understanding

Existing behavior analysis methods for scene understanding typically includes two key steps: establishing behavior models from training samples and matching test samples with established models [18, 19], where typical behavior models are usually obtained by clustering trajectory data. In traffic monitoring scenarios, combined with scene knowledge, the corresponding semantic interpretation of behaviors can be given, such as heading straight, turning left, turning right, U-turn, converse driving, and illegal lane changing [20–22]. Since the quality of trajectory-based behavior analysis methods is highly dependent on robust tracking of vehicles, there is a strong interest among researchers to develop methods for modeling behavior without explicitly tracking objects (or at least without high-precision trajectories). Probabilistic topic models (PTM), such as probabilistic Latent Semantic Analysis (pLSA) [23], Hierarchical Dirichlet Processes (HDP) [24], Latent Dirichlet Allocation (LDA) [25], Cas-LDA [26], and Dual-HDP and Dynamic Dual-HDP [6, 7] treat low-level features as visual words in video sequences, which are segmented into clips that represent documents. The learned topics are shared by all documents and indicate the dominant motion patterns in the scene. However, all these studies were conducted within a single scene or a group of adjacent/similar scenarios, and the trained models cannot be migrated well across domains. The latter category is most relevant to our approach, since we also use LDA topic model [25] to learn topics/activities in a surveillance scene. However, instead of directly matching the test samples in the target domain with the models learned from the source domain, in our approach, we first combine the Word2Vec word vector model and LDA topic model to construct the topic-word vector matrix and then use the text convolutional neural networks (TextCNN) with two superimposed convolutional layers to perform multilabel classification. This transforms the cross-domain traffic scene understanding problem into a multilabel classification problem to solve.

### 2.2. Multiscene Understanding

Activity modeling and anomalous event detection in multicamera networks is extremely challenging [27–29], regardless of whether the scenarios covered by these camera views overlap. The aim of these studies is to discover connections and correlations between multiple overlapping or nonoverlapping scenarios by sharing information between multiple camera views. This is orthogonal to our area of interest, where our study focuses more on knowledge migration between scenarios that do not have topologically connected regions.

Few methods have attempted to exploit the correlation between scenes without topological relationships [10–12, 30]. Xu et al. [10] used trajectory-based event descriptions and compared cross-domain trajectories using the Kullback-Leibler Divergence (KLD) between their mixtures of Gaussian (GMM) representation. Yoo et al. [11] reasonably assumed that the latent variables of the relationship are given (from the source domain to the target domain) and trained separately for each motion pattern. Then, a transfer learning scheme for the convex optimization problem is proposed using the geometric relationship between the motion patterns in the target scene and the motion patterns in the source scene. Shuai et al. [12] extracted the depth representations of traffic scene images by convolutional neural networks (CNN) to achieve cross-domain scene matching based on cross-domain dense correspondences and a probabilistic Markov random field (MRF). Khorkhar et al. [30] compared and matched two motion pattern mixture distributions by estimating the similarity transformation between the motion pattern mixture distributions in different scenarios. However, the above approaches can only transfer scene knowledge on the basis of comparing/quantifying cross-domain similarities between the entire domains, while neglecting the cross-domain similarity between those different but locally related scenarios. Our approach is to solve the cross-domain traffic scene understanding problem by transforming it into a multilabel classification problem with activity attributes (local motion patterns). When labeling the activity attributes of the target domain, there is no need to select the source domain, which avoids the phenomenon of performance degradation or even negative transfer due to wrong source domain selection. In addition, the Kullback-Leibler Divergence (KLD) is exploited to measure the similarity between probabilistically represented activities in different scenarios. Before the topic-word vector matrix of the target domain scenes is fed into TextCNN for multilabel classification, a topic-word vector matrix transformation strategy is executed to reduce cross-scene variance.

### 2.3. Deep Learning for Scene Understanding

CNN-based models perform extremely well in scene recognition tasks [12, 31, 32]. However, these models are not applicable to the problem of this paper because traffic scene images considered in our study have significant appearance variations due to being in cross-domain scenarios. Modified convolutional structure models for semantic scene segmentation have also obtained state-of-the-art results by learning to decode low-resolution image representations into pixel-level predictions [33–35]. These methods are also not directly applicable to surveillance scenarios, because the context of different surveillance scenarios is no longer stationary and they can differ significantly from each other. In our problem, the performance of these methods may degrade because the images of different scenarios have a great variation in appearance. It is unreliable to associate cross-domain scenarios based on the appearance of the scenarios; the important clue is the activity. In our approach, instead of directly using the trajectory points (positions and moving directions) of moving objects as input into TextCNN, we firstly learn topics (considered as activities) in the scene using Latent Dirichlet Allocation (LDA). Then, a topic-word vector matrix is constructed by combining the Word2Vec word vector model and the LDA topic model, which considers both word granularity level features and the expression of overall semantic features at the text granularity level.

## 3. Integrating LDA Topic Model and TextCNN

### 3.1. Topic-Word Vector Representation

We consider combining the Word2Vec word vector with the LDA topic vector to represent the text feature matrix at both word granularity and text granularity levels. Word2Vec mines the meaning of words at the word granularity level for fine-grained semantic representation of text. LDA constructs the topic distribution of text from the text granularity level by probabilistic model, focusing on the overall semantic representation of text.

#### 3.1.1. Learning Topics/Activities with Topic Model

Given a surveillance scene, we firstly learn activities in the scene using Latent Dirichlet Allocation (LDA) [25]. Similar to Dual-HDP and Dynamic Dual-HDP [6, 7], we also use topics to represent activities, and LDA generates a set of topics/activities to explain each scene. Then, each motion pattern in the scene is considered as a subset of activities.

Under our framework, a traffic video sample can be segmented into nonoverlapping clips with equal frame length. Video clips are treated as documents and the trajectory points (positions and moving directions) of moving objects are treated as motion words. At this point, we treat a trajectory within a video clip as a sentence. Each video clip contains multiple trajectories. Therefore, each video clip is mapped to a motion word bag or a collection of sentences and embodies one or more motion patterns. The positions and moving directions of trajectory points are computed as motion words which are quantized according to a codebook. The codebook uniformly quantizes the video frame into *N*_*a*_ × *N*_*b*_ small cells and the motion vector of objects into *N*_*m*_ fixed directions. Then, the size of the codebook is *N*_*v*_=*N*_*a*_ × *N*_*b*_ × *N*_*m*_.

**Figure 3:**
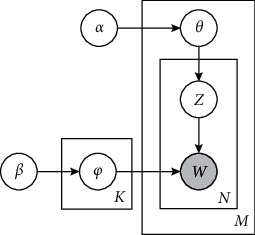
Graphical model for Latent Dirichlet Allocation.

The graphical representation of LDA model is shown in Figure 3. A traffic video captured in scene *s* is divided into M-segment video clips/documents. Each video clip is represented as a random mixture of *K* topics, where *K* represents the number of topics. Each topic is modeled as a polynomial distribution *φ*_*k*_=[*φ*_*k*1_, *φ*_*k*2_,…, *φ*_*kV*_], i.e., a mixture ratio *φ* ~ Dirichlet(*β*) of various motion words in the codebook.

Learning LDA for scene *s* is to discover the K topics (activities), expressed as polynomial parameters *φ*_*k*_^*s*^. For video clip *j*, the polynomial distribution *θ*_*j*_=[*θ*_*j*1_, *θ*_*j*2_,…, *θ*_*jK*_] over the K topics is generated by the Dirichlet distribution Dirichlet(*θ*_*j*_*|α*). For each motion word *w*_*ji*_ in video clip *j*, the topic *z*_*ji*_=*k* is determined by probability parameter *θ*_*jk*_; *φ*_*z*_*ji*__ determines the generation of motion word *w*_*ji*_.

Given *α* and *β*, the joint distribution of topic mixture parameter *θ*, motion word mixture parameter *φ*, topics *z*_*j*_={*z*_*ji*_}, and motion words *w*_*j*_={*w*_*ji*_} is

(1) $$\begin{array}{c} p\left(\theta_{j},z_{j},\varphi ,w_{j}|\alpha ,\beta \right)=p\left(\theta_{j}|\alpha \right)p\left(\varphi |\beta \right)\prod_{i=1}^{N_{j}}p\left(z_{ji}|\theta_{j}\right)p\left(w_{ji}|z_{ji},\varphi \right), \end{array}$$

where *N*_*j*_ is the number of motion words in the video clip *j*.

The Gibbs Sampling algorithm [36] is employed for parameter estimation. After sampling converges, the document-topic distribution matrix *θ* and the topic-word distribution matrix *φ* are generated. Each video clip *j* is now represented as a topic profile *θ*_*j*_^*s*^, and scene *s* can be represented by its constituent activities *φ*_*k*_^*s*^.

#### 3.1.2. Constructing Topic-Word Vector

In this work, we encode the motion words using Skip-Gram training model of Word2Vec [37] to form low-dimensional, dense word vectors. The documents/video clips in the document set/video are mapped over the pretrained word vectors to form a word vector matrix.

When modeling at the text granularity level, instead of directly applying the LDA model to map documents to the dimension of topics, documents are extended with features based on the LDA topic probability maximization principle to construct a document representation model that integrates word vectors with LDA. Using the maximum text length of documents in the document set as the reference, the document-topic distribution matrix is used to find the topic with the highest probability corresponding to the document. Then, after finding the word probability distribution under this topic through the topic-word distribution matrix, words are mapped into word vectors according to their probabilities from high to low, and the word vector matrix is filled in order until the number of rows of the constructed word vector matrix is equal to the maximum text length.

### 3.2. LDA-TextCNN Architecture

An overview of the proposed framework for cross-domain traffic scene understanding is depicted in Figure 2. After constructing the word vector matrix and topic vector learning (Figure 2(a)), the word vector matrix is populated based on the LDA topic probability maximization principle to complete the construction of the topic-word vector matrix (Figure 2(b)). The dimensionality of the topic-word vectors is denoted by *d*. In order to make the text feature extraction depth of TextCNN model wider and the classification effect more accurate, two convolutional layers are superimposed on the basic TextCNN model [38, 39].

When extracting the local features of the topic-word vector matrix in the first convolutional layer, we use three filter region sizes: 3, 4, and 5 and set the number of feature maps for each region size to 100 (Figure 2(c)). Then, the vectors obtained through the first convolution layer are fused and concatenated to form the concatenated feature vector matrix (Figure 2(d)). This concatenation greatly enriches the semantic feature representation of documents. When performing quadratic convolution (Figure 2(e)) on the concatenated feature vector matrix, we set three filter region sizes: 7, 8, and 9, each of which also has 100 feature maps. Corresponding to the shallow features obtained by the first convolution, the features obtained at this time are considered as deep features.

In the pooling layer, we perform 1-max pooling over each deep feature map, generating a feature vector of length 1 for each filter (Figure 2(f)). That is, the maximum value of each feature vector is extracted to represent the feature vector, and the maximum value is considered to represent the most important feature. After 1-max pooling for all the feature vectors, each maximum value needs to be cascaded to get the final feature vector of the pooling layer.

The feature vectors cascaded after the pooling layer are fully connected with the label set |*L*| neurons as the output layer. The sigmoid function is adopted as the output function. To avoid overfitting the training set, we use the dropout method proposed by Srivastava et al. [40]. Set a certain percentage of the neuron weights in the hidden layer to not work, thus reducing the computational effort and also avoiding the overfitting phenomenon during the training process to some extent. The time complexity is *O*(∑_*l*=1_^*L*^*W*_*l*_*H*_*l*_*D*_*l*_^2^*Z*_*l*_), which is the accumulation of the time complexity of the two convolutional layers, where *l* is the *l*th convolutional layer of TextCNN, *L* is the number of convolutional layers, *W* is the length of the input motion word sequence, *H* is the height of each filter, *D* is the dimensionality of the topic-word vectors, *Z* the number of sizes of filters, and the size of filters (feature maps) is also *D*. The space complexity is O *O*(∑_*l*=1_^*L*^*W*_*l*_*D*_*l*_*Z*_*l*_), and the key lies in the feature maps output from each convolutional layer.

## 4. Cross-Domain Transfer

We are ultimately interested in cross-domain migration of local prior knowledge, facilitating the cross-domain scene understanding by mapping object's activity attribute labels. After the above topics/activities learning using LDA, each topic/activity is modeled as a polynomial distribution *φ*_*k*_ of various motion words in the codebook. This is derived from the intuition that two activities which appear semantically similar to humans should have a similar distribution of motion words. From the transferability measurement perspective alone, an obvious alternative is to find rigid similarity transformations of each activity in the target domain scene to the source domain scene separately as a basis for activity attribute labels propagation. However, this would be overfitting, because, in this case, any activity model can be easily transformed into the same target activity model by the transformation matrix, which makes it difficult to choose the appropriate activity attribute labels for the target domain scene to be labeled. In contrast, our proposed method is tractable and free from overfiltering since it takes into account the full distribution of activities in the scene and feeds the transformed topic-word vector matrix of the target domain scene into the aforementioned TextCNN for multilabel classification.

We next address how to align two scenarios (source domain scene and target domain scene) and transform the topic-word vector matrix of target domain scene. To quantify the similarity between probabilistically represented activities in source domain scene and target domain scene, we exploit the Kullback-Leibler Divergence [30] (KLD) to measure the distance between distributions. More specifically, given *K*_*s*_ topics/activities {*φ*_*k*_*s*__^*s*^}_*k*_*s*_=1_^*K*_*s*_^ in source domain scene *s* and *K*_*t*_ topics/activities {*φ*_*k*_*t*__^*t*^}_*k*_*t*_=1_^*K*_*t*_^ in target domain scene *t*, the distance between topic/activity *φ*_*k*_*s*__^*s*^ and topic/activity *φ*_*k*_*t*__^*t*^ is defined as *D*_KL_ in

(2) $$\begin{array}{c} D_{\text{KL}}\left(\varphi_{k_{s}}^{s}\left\|\;\right.\varphi_{k_{t}}^{t}\right)=\sum_{v=1}^{N_{v}}\varphi_{k_{s}}^{s}\cdot \;\log\left(\frac{\varphi_{k_{s}}^{s}}{\varphi_{k_{t}}^{t}}\right). \end{array}$$

**Figure 4:**
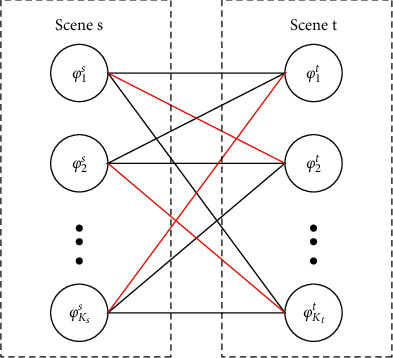
Weighted bipartite graph matching.

Then, we convert the problem of correspondence between the activities within the source domain scene and target domain scene into the weighted bipartite graph matching problem and solve it using the Kuhn-Munkres (KM) algorithm. By giving each vertex a top mark, the algorithm transforms the maximum weight matching into a continuous searching for an augmentation path to find a perfect matching [41, 42]. The matching principle is shown in Figure 4.

Topics/activities in the source domain scene and the target domain scene are regarded as the left vertexes and right vertexes of bipartite graph respectively. The number of left vertexes is the number of topics/activities in the source domain scene, and the number of right vertexes is the number of topics/activities in the target domain scene. The Kullback-Leibler Divergence *D*_KL_(*φ*_*k*_*s*__^*s*^‖ *φ*_*k*_*t*__^*t*^) is calculated as the weight of the edge connected by the left and right vertex. Through weighted bipartite graph matching, the correspondence between topics/activities in the two scenarios is obtained. This is necessary because the correspondence cannot be estimated unless the activities in the two scenarios are aligned.

For wide-area surveillance, semantically equivalent activities differ only in the geometry of their views but are equivalent under the geometric similarity transformation H (a 3 × 3 matrix). We denote the transformation matrix *H* as

(3) $$\begin{array}{c} H=\left[\begin{array}{ccc} s_{x} & 0 & t_{x}\\ 0 & s_{y} & t_{y}\\ 0 & 0 & 1 \end{array}\right], \end{array}$$

where (*s*_*x*_, *s*_*y*_) and (*t*_*x*_, *t*_*y*_) are the scaling and translation parameters, respectively. Considering that rotation transformation may change the activity attribute labels of the output, we did not consider rotation parameters in the geometric transformation.

To avoid overfitting, we consider that activities in the same scene share the same geometric transformation. Therefore, our goal is to find H which transforms *φ*_*k*_*t*__^*t*^ to *φ*_*k*_*t*__^*t* ′^ and to minimize the sum of the weights of the edges while keeping the corresponding relationship of the activities in the two scenarios unchanged. Then, we apply an objective function to find *H* as

(4) $$\begin{array}{c} \sum D_{\text{KL}}\left(\varphi_{k_{s}}^{s}\left\|\;\right.\varphi_{k_{t}}^{t}\right)=\underset{H}{\min}\sum_{k_{s}=1}^{K_{s}}\sum_{k_{t}^{\prime }=1}^{K_{t}}\sum_{v=1}^{N_{v}}\varphi_{k_{s}}^{s}\cdot \;\log\left(\frac{\varphi_{k_{s}}^{s}}{{\left(\varphi_{k_{t}^{\prime }}^{t}\right)}^{\prime }}\right), \end{array}$$

where (*φ*_*k*_*t*_′_^*t*^)′ is transformed from *φ*_*k*_*t*_′_^*t*^ by matrix *H*. *φ*_*k*_*t*_′_^*t*^ is the activity in target domain scene *t* that corresponds to activity *φ*_*k*_*s*__^*s*^ in source domain scene *s*.

This transformation is applied to topics/activities in a similar way to image transformation. That is to say, assume that *φ*_*k*_*t*__^*t*^ is a *N*_*a*_ × *N*_*b*_ × *N*_*m*_ matrix and (*φ*_*k*_*t*__^*t*^)′ is transformed from *φ*_*k*_*t*__^*t*^ through transformation matrix *H*. We first need to estimate the size *N*_*a*_′ × *N*_*b*_′ × *N*_*m*_ of transformed topic/activity (*φ*_*k*_*t*__^*t*^)′ by *N*_*a*_′=*N*_*a*_ × *s*_*x*_ and *N*_*b*_′=*N*_*b*_ × *s*_*y*_. In order to obtain the value of each motion word in (*φ*_*k*_*t*__^*t*^)′, we need to trace back to the value of the corresponding motion word in the original topic/activity *φ*_*k*_*t*__^*t*^. It is worth noting that since the rotation parameter is not taken into account in the geometric transformation, we consider that the motion direction remains unchanged throughout the procedure of transformation. Some common interpolation methods can be used for this scaling task, including nearest neighbor interpolation, bilinear interpolation, and bicubic interpolation. In our present work, bicubic interpolation method is adopted.

After interpolation, the value of each pixel (*x*′, *y*′) is calculated exactly. However, since this transformation involves translation, the transformed topic/activity may extend beyond the original topic/activity boundary. That is, the scaled scene is beyond the boundary of the original scene and the codebook is inconsistent. To ensure that all topics/activities maintain the same codebook for comparability, for the transformed (*φ*_*k*_*t*__^*t*^)′, we keep only those parts that lie within the *N*_*a*_ × *N*_*b*_ range of the original topic/activity *φ*_*k*_*t*__^*t*^. After the above operation, the codebook of the transformed target domain scene and the original target domain scene remains unchanged. Meanwhile, the size of the transformed topic/activity (*φ*_*k*_*t*__^*t*^)′ is the same as that of the original topic/activity *φ*_*k*_*t*__^*t*^, *N*_*a*_ × *N*_*b*_ × *N*_*m*_. Finally, we obtain the transformed topic-word vector matrix (see Section 3.1), which is fed into the above TextCNN for multilabel classification (see Section 3.2).

## 5. Experimental Evaluation

### 5.1. Datasets and Settings

**Figure 5:**
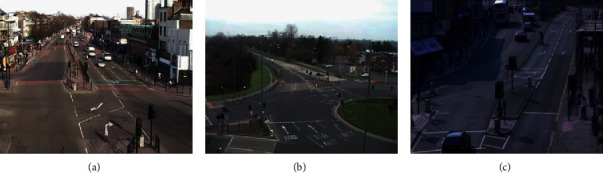
Sample frames for each scene: (a) Junction dataset; (b) Roundabout dataset; (c) Junction 2 dataset.

To verify the effectiveness of the proposed method, we conduct experiments on three different traffic videos [8, 26]. The three datasets were collected from three different real-world public road surveillance scenarios featured with large numbers of moving objects exhibiting typical traffic patterns well. Junction dataset: this dataset contains 60 minutes (90000 frames) of 25 fps video of a busy urban road junction with a frame size of 360 × 288 pixels. Roundabout dataset: this dataset contains about 60 minutes (90000 frames) of 25 fps video of a traffic Roundabout with a frame size of 360 × 288 pixels. Junction 2 dataset: this dataset contains 52 minutes (78000 frames) of 25 fps video of a busy urban road junction with a frame size of 360 × 288 pixels. Sample frames for each scene are shown in Figure 5.

Traffic video of each surveillance scene is segmented into nonoverlapping clips with equal length of 200 frames. As a result, 450, 450, and 390 clips are generated for Junction dataset, Roundabout dataset, and Junction 2 dataset, respectively. In order to build the motion dictionary, the 360 × 288 surveillance scene is quantized into 40 × 32 cells of size 9 × 9 and the moving directions are quantized into four directions perpendicular to each other. So, the size of the dictionary is 40 × 32 × 4.

Set the model parameters *α* = *K*/50, *β* = 0.01. Topics/activities are learned from each traffic video independently using LDA with *K*=20 topics/activities for each scene. We provide each video clip with a set of binary activity attribute labels (each label represents the activity of some objects in the scene), as shown in Table 1. Each unique combination of activity attributes present in the labeled clips reflected a scene-level behavior category.

In order to evaluate the performance of our proposed T-LDA-TextCNN method, we conducted two experiments: single-source domain transfer and multisource domains transfer. We compare the proposed T-LDA-TextCNN with the eight state-of-the-art baseline methods: (1) domain adaptation support vector machine (DASVM) [43]; (2) multidomain adaptation with heterogeneous sources (MDA-HS) [44]; (3) transfer kernel learning (TKL) [45]; (4) Discriminative Distribution Alignment (DDA) [46]; (5) stratified transfer learning (STL) [47]; (6) transfer neural network for activity recognition (TNNAR) [48]; (7) multiple feature spaces adaptation network (MFSAN) [49]; and (8) conditional weighting adversarial network (CWAN) [50].

### 5.2. Single-Source Domain Transfer

In this subsection, we evaluate the capability of our framework to transfer active attribute labels in the single-source domain case. We does not fix Junction dataset, Roundabout dataset, and Junction 2 dataset in turn as source domain datasets because transferring different activity attributes between different scenarios does not have reasonable feasibility in real applications. Therefore, we only use Junction dataset as source domain dataset because it has contained almost all the traffic patterns of both Roundabout dataset and Junction 2 dataset. We compare T-LDA-TextCNN with various kinds of single-source domain adaptation methods, including DASVM [43], TKL [45], STL [47], and TNNAR [48]. During experiments, the source domain selection procedure in these methods is not considered. In addition, our proposed T-LDA-TextCNN method is also compared with the LDA-TextCNN method without the proposed geometric transformation scheme.

**Figure 6:**
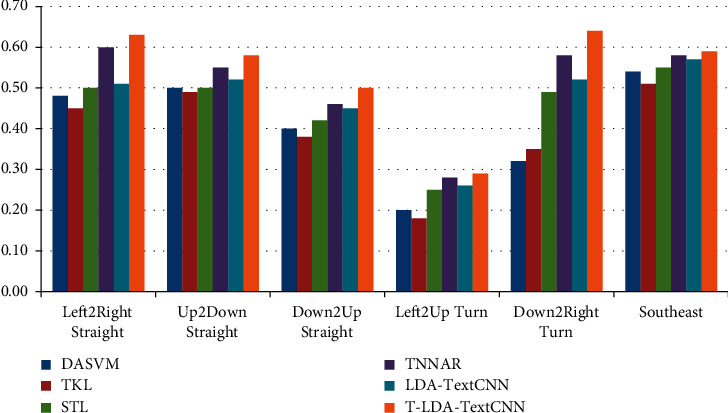
Precision of different approaches under single-source domain on Roundabout dataset.

**Figure 7:**
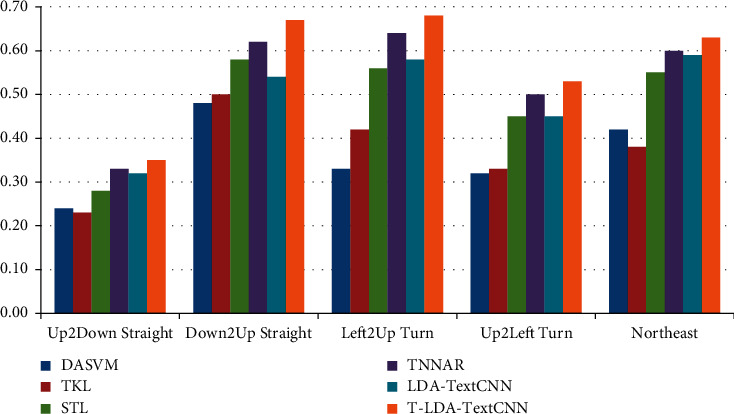
Precision of different approaches under single-source domain on Junction 2 dataset.

Two different comparative experiments are set up to verify the validity of the proposed method. In one case, video data of the target domain does not participate in the training at all, so as to illustrate the performance of T-LDA-TextCNN method under unsupervised learning. Precision is used as evaluation criterion for activity attribute annotation in target domain datasets (Roundabout dataset and Junction 2 dataset). Figures 6 and 7 show the transfer learning ability of T-LDA-TextCNN, DASVM, TKL, STL, TNNAR, and LDA-TextCNN on each activity attribute. These activity attributes are the intersection of the activity attributes of the source and target datasets. The proposed T-LDA-TextCNN not only significantly outperforms these four baseline methods, but also outperforms the LDA-TextCNN method without the proposed geometric transformation scheme.

**Figure 8:**
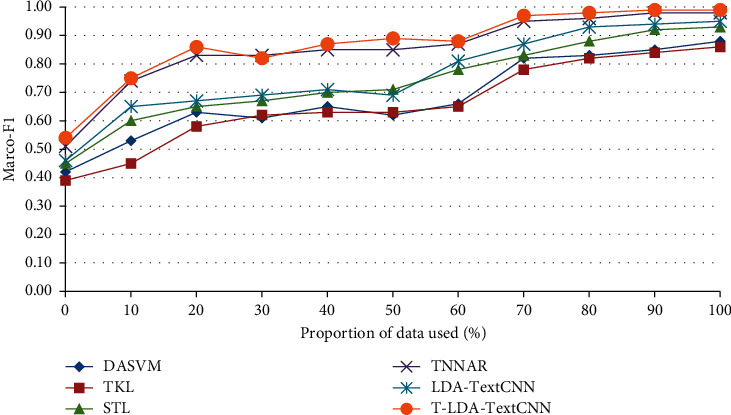
Marco-F1 of different approaches under single-source domain on Roundabout dataset.

**Figure 9:**
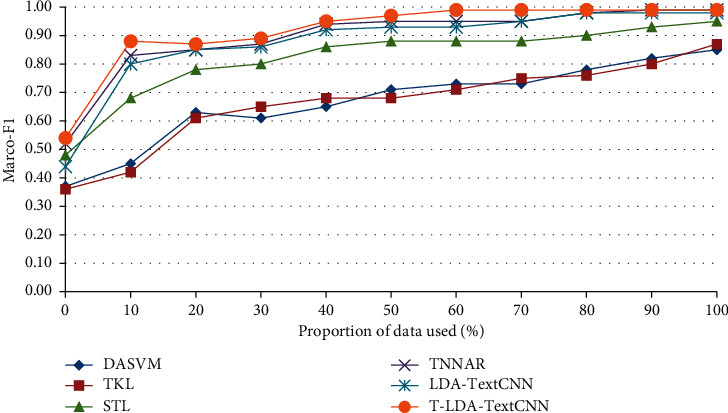
Marco-F1 of different approaches under single-source domain on Junction 2 dataset.

In the second comparison experiment, some labeled target domain video data are needed to assist in training the classifier, so as to illustrate the performance of T-LDA-TextCNN method under a small amount of labeled target domain video data training. The performance has been measured by Marco-F1 which is the mean of F1-measure of all activity attributes. Figures 8 and 9 plot the increment degree of Marco-F1 of different approaches versus the number of training samples on Roundabout dataset and Junction 2 dataset. Although LDA-TextCNN achieves higher performance than DASVM, TKL, and STL, T-LDA-TextCNN has greater improvement. The results clearly show that T-LDA-TextCNN significantly improves the performance of cross-domain activity recognition. The performance of deep neural network-based methods (T-LDA-TextCNN, TNNAR, and LDA-TextCNN) outperforms other methods (STL, TKL, and DASVM) across the board, which suggests that the structure of neural networks facilitates transfer learning, because hyperparameters can be easily shared across domains. In addition, T-LDA-TextCNN converges faster than LDA-TextCNN. This implies that the proposed geometric transformation scheme makes a significant contribution to the improvement of performance.

### 5.3. Multisource Domains Transfer

In practical situations, there are often multiple source domain scenarios related to the target domain scene. They have more or less knowledge (active attributes) related to the target domain scene, which can be utilized to improve the performance of the target classifier. In the experiments in this subsection, the source domain scenarios are no longer single, but multiple source domain scenarios related to the target domain scene. Roundabout dataset and Junction 2 dataset are fixed as the target domain dataset in turn. Junction dataset and Junction 2 dataset serve as the source domain datasets for Roundabout dataset, while Junction dataset and Roundabout dataset serve as the source domain datasets for Junction 2 dataset. The experimental setup and performance evaluation criteria are the same as those in Subsection 5.2, except that those baseline methods are replaced with multisource unsupervised domain adaptation methods, including MDA-HS [44], DDA [46], MFSAN [49], and CWAN [50]. Note that, in the T-LDA-TextCNN method, the topic-word vector matrix of the second source domain scene needs to be geometrically transformed to align the two scenarios (the first source domain scene and the second source domain scene) before being fed into the TextCNN for training.

**Figure 10:**
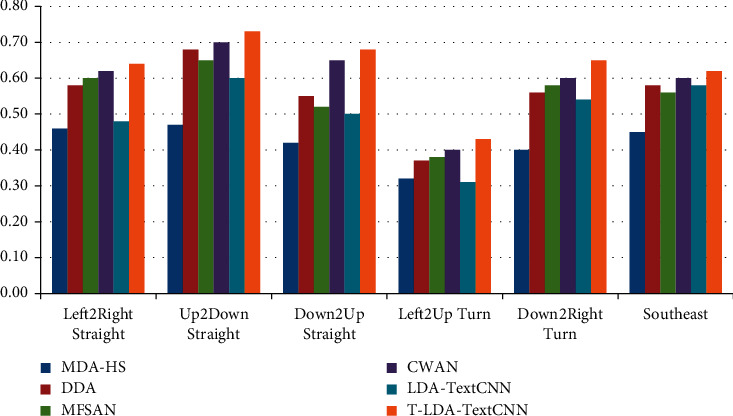
Precision of different approaches under multisource domain on Roundabout dataset.

**Figure 11:**
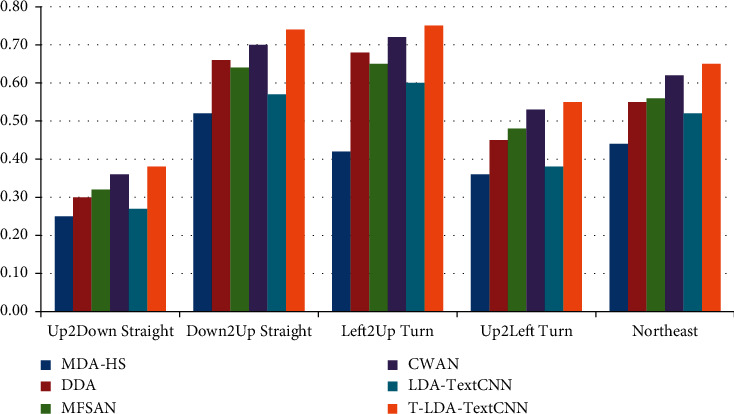
Precision of different approaches under multisource domain on Junction 2 dataset.

**Figure 12:**
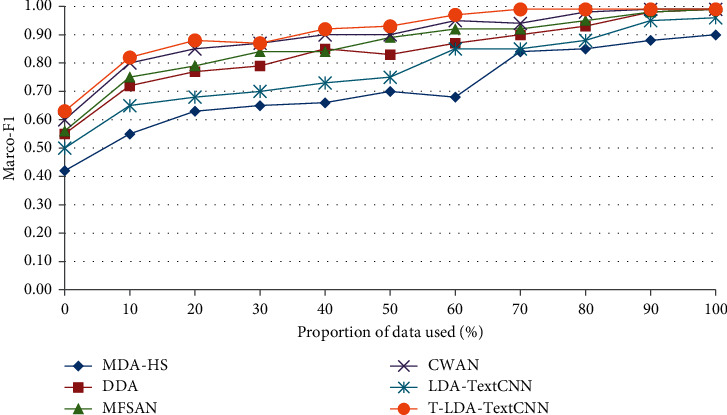
Marco-F1 of different approaches under multisource domain on Roundabout dataset.

**Figure 13:**
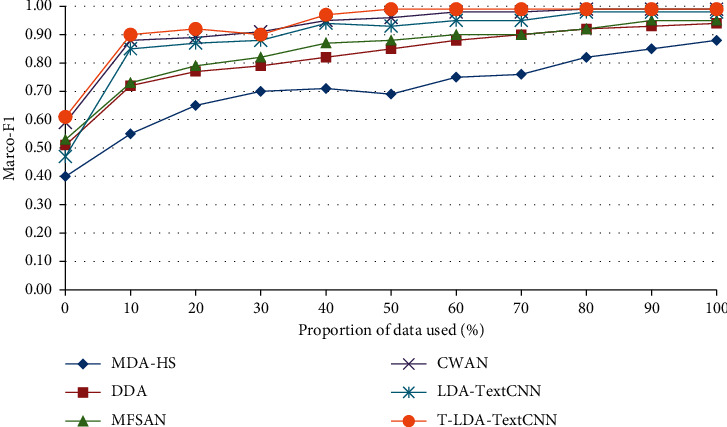
Marco-F1 of different approaches under multisource domain on Junction 2 dataset.

As shown in Figures 10 and 11, the precision of the annotation for each activity attribute increases to different degrees, among which the annotation precision of Up2Down Straight, Down2Up Straight, and Left2Up Turn increases more. All three activity attributes are present in the first source domain dataset, the second source domain dataset, and the target domain dataset. Figures 12 and 13 illustrate the performance of different methods under the auxiliary training with labeled target domain video data, and Marco-F1 values increase to some extent. In conclusion, the overall effect of knowledge transfer using multiple source domains is better than that of single-source domains. The performance of T-LDA-TextCNN outperforms the other methods (MDA-HS, DDA, MFSAN, CWAN, and LDA-TextCNN) across the board. However, the effect of transfer learning does not increase in the same magnitude incrementally. In the evaluation experiment with Roundabout dataset as the target domain dataset, the annotation accuracy for active attributes Up2Down Straight, Down2Up Straight, and Left2Up Turn increased by 25.86%, 36%, and 48.28%, respectively. The annotation accuracy for active attributes Left2Right Straight, Down2Right Turn, and Southeast increased by only 1.59%, 1.56%, and 5.08%, respectively. The evaluation experiment with Junction 2 dataset as the target domain dataset also has similar results. This is because the effect of transferring knowledge from source domain to target domain depends on the correlation between domains (overall or local). The greater the correlation is, the more meaningful the transferred knowledge is.

### 5.4. Impact of Different Optimizers on Performance

**Figure 14:**
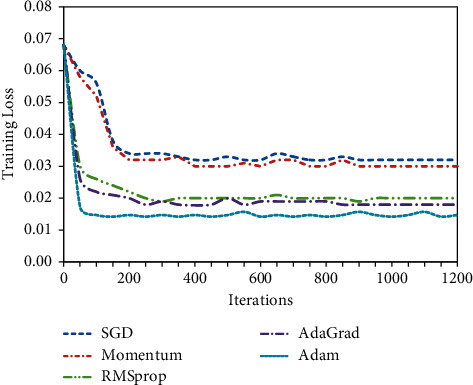
Training loss of T-LDA-TextCNN.

In this subsection, we evaluate the impact of SGD [51], AdaGrad [52], RMSprop [53], Momentum [54], and Adam [55] optimizers on the model training process. The experimental setup and performance evaluation criteria are the same as those in Subsection 5.2. Video data of the target domain are not involved in training at all to illustrate the performance of the T-LDA-TextCNN method using different optimizers under unsupervised learning. When comparing different optimization algorithms, we choose the same initial learning rate of 0.001. After the training of each optimizer, the activity attribute annotation precision of T-LDA-TextCNN method on target domain datasets (Roundabout dataset and Junction 2 dataset) is shown in Tables 1 and 2 respectively. The loss curves of different optimizers on the training set (source domain dataset) are shown in Figure 14.

It can be seen from Tables 2 and 3 and Figure 14 that, after the training of each optimizer, Adam optimizer has the highest activity attribute annotation precision and the fastest convergence speed compared with SGD, Momentum, RMSprop, and AdaGrad optimizers. The T-LDA-TextCNN method proposed in this paper adopts Adam optimizer.

### 5.5. Discussions

In this work, we focus on addressing the cross-domain traffic scene understanding problem, which substantially is distinguished from other cross-domain transfer methods on the following grounds.

1. DASVM method can only handle the case where the source and target domain features are isomorphic. After the source-domain samples are used to initialize the discriminant function for the target domain problem, DASVM method iteratively removes the source-domain samples and gradually adjusts the discriminant function to fit the target domain instances. Each iteration of DASVM takes a time equivalent to the time required for supervised SVM learning. TKL method is a data-dependent spectral learning method. However, TKL only adapts the edge distribution, ignoring the alignment of the conditional probability distribution. Therefore, more in-depth research on the joint distribution adaptation based on spectral learning is needed. However, when solving the multisource domains transfer problem, they all need to involve new learnable parameters, and the contribution of each source domain cannot be quantified.
2. STL and TNNAR methods are designed to solve cross-domain activity recognition (CDAR). STL exploits the intra-affinity of classes to iteratively perform intraclass knowledge transfer. TNNAR performs knowledge transfer for activity recognition through multisource domain selection and deep neural network. The source and target domain data of CDAR have the same dimensions, the same labels, and even the data distribution which can be the same. However, the scene is not reasonably feasible for practical cross-domain traffic scene understanding applications.
3. MDA-HS method trains the respective independent SVM classifiers based on the training data of each source domain. The final prediction for each target domain sample is then obtained by averaging the predictions of all classifiers. The computational complexity is dominated by SVM classifiers training. The samples of different source domains of the problem solved by MDA-HS method are represented by different types of features, while the target domain samples have all types of features. DDA method simultaneously performs classifier adaptation, distribution alignment, and distinguished embedding. Data from different domains have different feature representations. MFSAN method uses multiple domain-invariant representations to train multiple domain-specific classifiers and align the target sample outputs of the domain-specific classifiers. The training process fine-tunes all convolutional and pooling layers and trains classifier layers by backpropagation, which is computationally expensive. CWAN is a deep learning model for the multisource heterogeneous domain adaptation (MHDA) problem. Not only the importance of different source domains is quantified, but also the conditional distribution between source and target domains is used for knowledge transfer. However, when more source domains are involved, they also need to solve new learnable parameters, which will lead to complex optimization problems.

## 6. Conclusions

In this paper, we propose a novel framework for cross-domain traffic scene understanding by integrating deep learning and topic model. It transforms the cross-domain traffic scene understanding problem into a multilabel classification problem with activity attributes for solving. No source domain selection is required, avoiding performance degradation or even negative migration due to incorrect source domain selection. The Word2Vec word vector model and the LDA topic model are combined in the construction of the topic-word vector matrix, which takes into account the representation of both word granularity level features and overall semantic features at the text granularity level. The proposed scene alignment method is tractable and free from overfiltering due to the consideration of the full distribution of activities in the scene and feeding the transformed topic-word vector matrix of the target domain scene into the aforementioned TextCNN for multilabel classification. In future work, we aim to further investigate fully incremental transfer learning method in an online manner and apply this framework to environmental perception.

## Figures and Tables

**Table 1:** Activity attribute labels for different datasets.

Activity attribute labels	Junction dataset	Roundabout dataset	Junction 2 dataset
Left2Right Straight	✓	✓	-
Right2Left Straight	✓	-	-
Up2Down Straight	✓	✓	✓
Down2Up Straight	✓	✓	✓
Left2Up Turn	✓	✓	✓
Left2Down Turn	✓	-	-
Right2Up Turn	✓	-	-
Right2Down Turn	-	-	-
Up2Left Turn	✓	-	✓
Up2Right Turn	✓	-	-
Down2Left Turn	-	✓	-
Down2Right Turn	✓	✓	-
Up2Left U-turn	✓	-	-
Down2Right U-turn	✓	-	-
Southeast	✓	✓	-
Southwest	-	-	✓
Northwest	-	✓	-
Northeast	✓	-	✓

**Table 2:** Precision of different optimizers on Roundabout dataset.

Activity attributes	SGD	AdaGrad	RMSprop	Momentum	Adam
Left2Right Straight	0.49	0.58	0.56	0.50	0.63
Up2Down Straight	0.42	0.52	0.51	0.44	0.58
Down2Up Straight	0.34	0.45	0.43	0.36	0.50
Left2Up Turn	0.19	0.25	0.23	0.20	0.29
Down2Right Turn	0.40	0.55	0.50	0.42	0.64
Southeast	0.40	0.53	0.51	0.43	0.59

**Table 3:** Precision of different optimizers on Junction 2 dataset.

Activity attributes	SGD	AdaGrad	RMSprop	Momentum	Adam
Up2Down Straight	0.21	0.31	0.30	0.22	0.35
Down2Up Straight	0.39	0.60	0.61	0.42	0.67
Left2Up Turn	0.41	0.59	0.57	0.40	0.68
Up2Left Turn	0.36	0.45	0.42	0.38	0.53
Northeast	0.35	0.50	0.45	0.41	0.63

## Data Availability

The QMUL Junction dataset is available online at https://personal.ie.cuhk.edu.hk/∼ccloy/downloads_qmul_junction.html. The QMUL Roundabout dataset is available online at https://www.eecs.qmul.ac.uk/∼sgg/QMUL_Junction_Datasets/Roundabout/Roundabout.html. The QMUL Junction 2 dataset is available online at https://www.eecs.qmul.ac.uk/∼sgg/QMUL_Junction_Datasets/Junction2/Junction2.html.
